# Protocol - realist and meta-narrative evidence synthesis: Evolving Standards (RAMESES)

**DOI:** 10.1186/1471-2288-11-115

**Published:** 2011-08-16

**Authors:** Trisha Greenhalgh, Geoff Wong, Gill Westhorp, Ray Pawson

**Affiliations:** 1 Healthcare Innovation and Policy Unit, Centre for Primary Care and Public Health, Blizard Institute, Barts and The London School of Medicine and Dentistry, London E1 2AB, UK; 2 Community Matters, PO Box 443, Mt Torrens, South Australia, SA 5244, Australia; 3 School of Sociology and Social Policy, University of Leeds, Leeds, LS2 9JT, UK

**Keywords:** systematic review, realist review or synthesis, meta-narrative review

## Abstract

**Background:**

There is growing interest in theory-driven, qualitative and mixed-method approaches to systematic review as an alternative to (or to extend and supplement) conventional Cochrane-style reviews. These approaches offer the potential to expand the knowledge base in policy-relevant areas - for example by explaining the success, failure or mixed fortunes of complex interventions. However, the quality of such reviews can be difficult to assess. This study aims to produce methodological guidance, publication standards and training resources for those seeking to use the realist and/or meta-narrative approach to systematic review.

**Methods/design:**

We will: [a] collate and summarise existing literature on the principles of good practice in realist and meta-narrative systematic review; [b] consider the extent to which these principles have been followed by published and in-progress reviews, thereby identifying how rigour may be lost and how existing methods could be improved; [c] using an online Delphi method with an interdisciplinary panel of experts from academia and policy, produce a draft set of methodological steps and publication standards; [d] produce training materials with learning outcomes linked to these steps; [e] pilot these standards and training materials prospectively on real reviews-in-progress, capturing methodological and other challenges as they arise; [f] synthesise expert input, evidence review and real-time problem analysis into more definitive guidance and standards; [g] disseminate outputs to audiences in academia and policy. The outputs of the study will be threefold:

1. Quality standards and methodological guidance for realist and meta-narrative reviews for use by researchers, research sponsors, students and supervisors

2. A 'RAMESES' (Realist and Meta-review Evidence Synthesis: Evolving Standards) statement (comparable to CONSORT or PRISMA) of publication standards for such reviews, published in an open-access academic journal.

3. A training module for researchers, including learning outcomes, outline course materials and assessment criteria.

**Discussion:**

Realist and meta-narrative review are relatively new approaches to systematic review whose overall place in the secondary research toolkit is not yet fully established. As with all secondary research methods, guidance on quality assurance and uniform reporting is an important step towards improving quality and consistency of studies.

## Background

### Introduction

Academics and policymakers are increasingly interested in 'policy-friendly' approaches to evidence synthesis which seek to illuminate issues and understand contextual influences on whether, why and how interventions might work [1-4]. A number of different approaches have been used to try to address this goal. Qualitative and mixed-method reviews are often used to supplement, extend and in some circumstances replace Cochrane-style systematic reviews [5-11]. Theory-driven approaches to such reviews include realist and meta-narrative review. Realist review was originally developed by Pawson for complex social interventions to explore systematically how contextual factors influence the link between intervention and outcome (summed up in the question "what works, how, for whom, in what circumstances and to what extent?") [12,13]. Greenhalgh et al. developed meta-narrative review as an adaptation of realist review, for use when a policy-related topic has been researched in different ways by multiple groups of scientists, especially when key terms have different meanings in different literatures [14].

Quality checklists and reporting standards are common (and, increasingly, expected) in health services research - see for example CONSORT for randomised controlled trials [15], AGREE for clinical guidelines [16], PRISMA for Cochrane-style systematic reviews [17] and SQUIRE for quality improvement studies [18]. They have two main purposes: they help researchers design and undertake robust studies, and they help reviewers and potential users of research outputs assess validity and reliability. This project seeks to produce a set of quality criteria and comparable reporting guidance for realist and meta-narrative reviews.

### What are realist and meta-narrative reviews?

Realist and meta-narrative reviews are systematic, theory-driven interpretative techniques, which were developed to help make sense of heterogeneous evidence about complex interventions applied in diverse contexts in a way that informs policy. Interventions have been described as "theory incarnate" [19], driven by hypotheses, hunches, conjectures and aspirations about individual and social betterment. Strengthening a review process that helps to sift and sort these theories may be an important step in producing better interventions.

Realist review seeks to unpack the relationships between context, mechanism and outcomes (sometimes abbreviated as C-M-O) - i.e. how particular contexts have 'triggered' (or interfered with) mechanisms to generate the observed outcomes [4]. Its philosophical basis is realism, which assumes the existence of an external reality (a 'real world') but one that is 'filtered' (i.e. perceived, interpreted and responded to) through human senses, volitions, language and culture. Such human processing initiates a constant process of self-generated change in all social institutions, a vital process that has to be accommodated in evaluating social programmes.

In order to understand how outcomes are generated, the roles of both external reality and human understanding and response need to be incorporated. Realism does this through the concept of mechanisms, whose precise definition is contested but for which a working definition is '...underlying entities, processes, or structures which operate in particular contexts to generate outcomes of interest.' [20]. Different contexts interact with different mechanisms to make particular outcomes more or less likely - hence a realist review produces recommendations of the general format "In situations [X], complex intervention [Y], modified in this way and taking account of these contingencies, may be appropriate". Realist reviews can be undertaken in parallel with traditional Cochrane reviews (see the complementary Cochrane and realist reviews of school feeding programmes in disadvantaged children [21,22]). The Cochrane review produced an estimate of effect size whilst the realist review addressed why and how school feeding programmes 'worked', explained examples of when they did not 'work', and produced practical recommendations for policymakers.

Meta-narrative review was originally developed by Greenhalgh et al. to try to explain the apparently disparate data encountered in their review of diffusion of innovation in healthcare organisations [14,23]. Core concepts such as 'diffusion', 'innovation', 'adoption' and 'routinisation' had been conceptualised and studied very differently by researchers from a wide range of primary disciplines including psychology, sociology, economics, management and even philosophy. Whilst some studies had been framed as the implementation of a complex intervention in a social context (thus lending themselves to a realist analysis), others had not. Preliminary questions needed to be asked, such as "what exactly did these researchers mean when they used the terms 'diffusion', 'innovation' and so on?", "how did they link the different concepts in a theoretical model - either as a context-mechanism-outcome proposition or otherwise?" and "what explicit or implicit assumptions were made by different researchers about the nature of reality?".

These questions prompted the development of meta-narrative review, which sought to illuminate the different paradigmatic approaches to a complex topic area by considering how the 'same' topic had been differently conceptualised, theorised and empirically studied by different groups of researchers. Meta-narrative review is particularly suited to topics where there is dissent about the nature of what is being studied and what is the best empirical approach to studying it. For example, Best et al., in a review of knowledge translation and exchange, asked how different research teams had conceptualised the terms 'knowledge', 'translation' and 'exchange' - and what different theoretical models and empirical approaches had been built on these different conceptualisations [24]. Thus meta-narrative review potentially offers another strategy to assist policy makers to understand and interpret a conflicting body of research, and therefore to use it more effectively in their work.

### The need for standards in theory-driven systematic reviews

Realist and meta-narrative approaches can capitalise on and help build common ground between social researchers and policy teams. Many researchers are attracted to these approaches because they allow systematic exploration of how and why complex interventions work. Policymakers are attracted to them because they are potentially able to answer questions relevant to practical decisions (not merely "what is the impact of X?" but "if we invest in X, to which particular sectors should we target it, how might implementation be improved and how might we maximise its impact?")

Whilst interest in such approaches is burgeoning, it is our experience that these approaches are sometimes being applied in ways that are not always true to the core principles set out in previous methodological guidance [4,13,25,26]. Some reviews published under the 'realist' banner are not systematic, not theory-driven and/or not consistent with realist philosophy. The meta-narrative label has also been misapplied in reviews which have no systematic methodology. For these reasons, we believe that the time has come to develop formal standards and training materials.

There is a philosophical problem here, however. Realist and meta-narrative approaches are interpretive processes (that is, they are based on building plausible evidenced explanations of observed outcomes, presented predominantly in narrative form), hence they do not easily lend themselves to a formal procedure for quality checking. Indeed, we have argued previously that the core tasks in such reviews are thinking, reflecting and interpreting [4,27]. In these respects, realist and meta-narrative reviews face a problem similar to that encountered in assessing qualitative research - namely the extent to which guidelines, standards and checklists can ever capture the essence of quality. Some qualitative researchers are openly dismissive of the 'technical checklist' approach as an assurance of quality in systematic review [28]. Whilst we acknowledge such views, we believe that from a pragmatic perspective, formal quality criteria - with appropriate caveats - are likely to add to, rather than detract from, the overall quality of outputs in this field. Scientific discovery is never the mere mechanical application of set procedures [29]. Accordingly, research protocols should aim to guide rather than dictate.

### The online Delphi method

This study will use the online Delphi method and in this section we introduce, explain and justify our use of this method. The essence of the Delphi technique is to engender reflection and discussion amongst a panel of experts with a view to getting as close as possible to consensus and documenting both the agreements reached and the nature and extent of residual disagreement [30]. It was used, for example, to set the original care standards which formed the basis of the Quality and Outcomes Framework for United Kingdom general practitioners [31]. Factors which have been shown to influence quality in the Delphi process include: [a] composition (expertise, diversity) of the expert panel; [b] selection of background papers and evidence to be discussed by that panel (completeness, validity, representativeness); [c] adequacy of opportunities to read and reflect (balance between accommodating experts' busy schedules and keeping to study milestones); [d] qualitative analysis of responses (depth of reflection and scholarship, articulation of key issues); [e] quantitative analysis of responses (appropriateness and accuracy of statistical analysis, clarity of presentation when this is fed back); and [f] how dissent and ambiguity are treated (e.g. avoidance of 'groupthink', openness to dissenting voices) [30,32,33].

Evidence suggests that the online medium is more likely to improve than jeopardise the quality of the consensus development process. Mail-only Delphi panels have been shown to be as reliable as face-to-face panels [34]. Asynchronous online communication has well-established benefits in promoting reflection and knowledge construction [35]. There are over 100 empirical examples of successful online Delphi studies conducted between geographically dispersed participants (see for example [33,36-40]). We have been unable to find any online Delphi study which identified the communication medium as a significant limitation. On the contrary, many authors described significant advantages of the online approach, especially when dealing with an international sample of experts. One group commented: *"Our online review process was less costly, quicker, and more flexible with regard to reviewer time commitment, because the process could accommodate their individual schedules." *[40].

Critical commentaries on the Delphi process have identified a number of issues which may prove problematic, for example *"issues surrounding problem identification, researcher skills and data presentation" *[30] or *"the definition of consensus; the issue of anonymity vs. quasi-anonymity for participants; how to estimate the time needed to collect the data, analyse each 'round', feed back results to participants, and gain their responses to this feedback; how to define and select the 'experts' who will be asked to participate; how to enhance response rates; and how many 'rounds' to conduct." *[33]. These comments suggest that it is the underlying design and rigour of the research process which is key to the quality of the study, and not the medium through which this process happens.

## Methods/design

### Research questions

1. What are the key steps in producing a valid and reliable systematic review using a realist or meta-narrative approach?

2. How might 'high' and 'low' quality in such reviews be defined and assessed [a] at the grant application stage; [b] during the review; [c] at publication stage and [d] by end-users of such reviews?

3. What are the key learning outcomes for a student of realist or meta-narrative review, and how might performance against these outcomes be assessed?

### Study design

**Figure 1:**
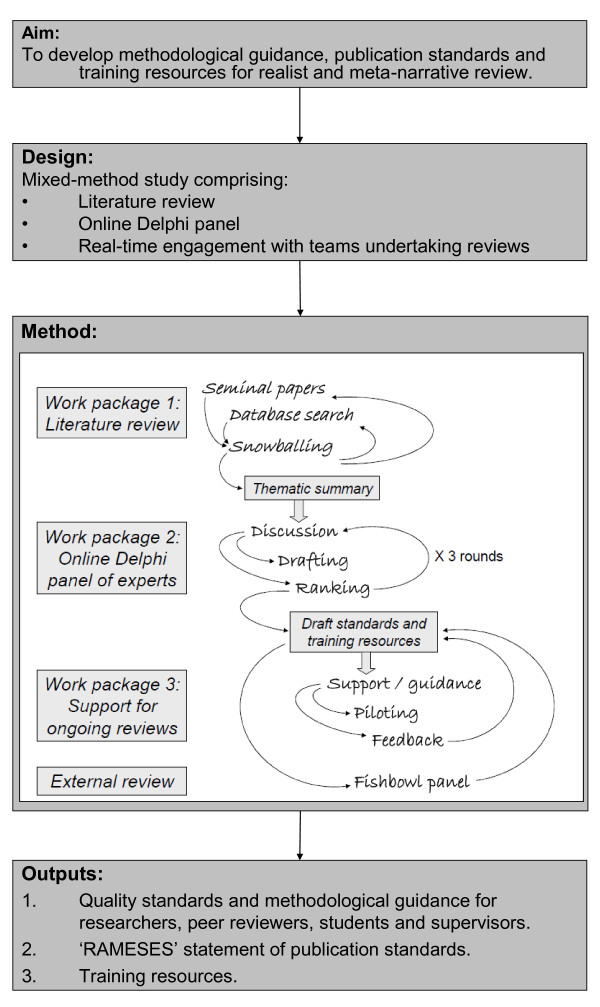
**Study protocol**.

Literature review, iterative online Delphi panel and real-time engagement with new, ongoing reviews (Figure 1).

### Study objectives

1. To collate and summarise the literature on the principles of good practice in realist and meta-narrative reviews, highlighting in particular how and why these differ from conventional forms of systematic review and from each other.

2. To consider the extent to which these principles have been followed by published and in-progress reviews, thereby identifying how rigour may be lost and how existing principles could be improved.

3. Using an online Delphi method with an interdisciplinary panel of experts from academia and policy, to produce, in draft form, an explicit and accessible set of methodological guidance and publication standards.

4. To produce training materials with learning outcomes linked to these steps and standards.

5. To pilot these standards and training materials prospectively on real reviews-in-progress, capturing methodological and other challenges as they arise.

6. To synthesise expert input, evidence review and real-time problem analysis into more definitive guidance and standards

7. To disseminate these guidance and standards to audiences in academia and policy

(1) and (2) will be achieved via a narrative review of the literature and supplemented by collating feedback from presentation(s) and workshop(s). These will feed into (3), which will be achieved via an online Delphi panel. The panel will include wide representation from researchers, students, policymakers, theorists and research sponsors. For (4), we will draw on our experience in developing and delivering relevant education modules. For (5), we will capture new realist reviews in progress as people approach us for help and guidance and seek their informed participation in piloting the new materials. (6) and (7) will be addressed by preparing academic publications, online resources and by delivering presentations and workshops.

### Intended outputs

We aim to generate three main outputs:

1. Quality standards and methodological guidance for realist and meta-narrative reviews for use by researchers, research sponsors, students and supervisors

2. A 'RAMESES' statement (comparable to CONSORT or PRISMA) of publication standards for such reviews, published in an open-access academic journal.

3. A training module for researchers, including learning outcomes, outline course materials and assessment criteria.

### Management and governance

The development of guidelines and guidance is a complex and contested process [41]. It is crucial to avoid the 'GOBSAT' (good old boys sat around a table) approach and ensure that [a] those who contribute to the process represent a diverse, informed and representative sample from both academia and policymaking and that [b] the process itself is systematic, auditable and justifiable. To that end, we will have a small core research team which will meet regularly to review progress, set the next work phase and produce minutes. We will report six-monthly to an advisory steering group, to whom we will present a project update and financial report.

In addition, approximately halfway through the study period, we will present our emerging findings formally to a panel of external researchers in order to collate additional feedback in a technique known as the 'fishbowl'. We will recruit a maximum variety sample of approximately 10 experts in systematic review. The main criterion for inclusion will be academic standing in the critical appraisal and evaluation of qualitative research studies and/or in evidence synthesis, including but not limited to those already familiar with realist or meta-narrative review. We will circulate materials in advance of the fishbowl workshop, including goals of the project, methodology and provisional standards and guidance. The fishbowl session will comprise presentation from the research team followed by discussion, facilitated by someone outside the core research team. The session will be recorded and minuted, and recommendations used to inform revision of the protocol as needed.

The study was deemed exempt from NHS research ethics approval (personal communication S Burke 14.2.11, East London and City Research Ethics Committee).

### Details of literature search methods

Our initial exploratory searches have found that the literature in this field is currently small but is expanding rapidly, and that it is of broad scope, variable quality and inconsistently indexed. The purpose of identifying published reviews is not to complete a census of realist and meta-narrative studies. Our comprehensive search will allow us to pinpoint real examples (or publications claiming to be examples) which provide rich detail on their usage of those review activities we wish to scrutinise and formalise. To that end, and drawing on a previous study which demonstrated the effectiveness and efficiency of the methods proposed [42], and employing the skills of a specialist librarian, we will employ three approaches:

1. Identifying seminal sources known to the research team and other experts in the field (e.g. via relevant networks and email lists).

2. Snowballing both backwards (pursuing references of references) and forwards (using citation-tracking software to identify subsequent publications citing the index paper) from seminal theoretical/methodological publications and empirical examples of realist and meta-narrative reviews. For reviews of heterogeneous bodies of evidence, snowball techniques are more effective and efficient than hand searching or using predefined search strings on electronic databases [42].

3. Database searching, especially with a view to identifying grey literature such as PhDs and unpublished reports (some will represent robust and critical applications of the methods and others will highlight 'commonly occurring mistakes and misconceptions').

In addition to identifying a broad range of examples of actual reviews, we will also capture papers describing methodological and theoretical critiques of the approaches being studied.

We will conduct a thematic analysis of this literature which will *initially *be oriented to addressing six questions, but to which we will add additional questions and topic areas (in order to better capture our analysis and understanding of the literature) as these emerge from our reading of the papers:

1. What are the strengths and weaknesses of realist and meta-narrative review from both a theoretical and a practical perspective?

2. How have these approaches actually been used? Are there areas where they appear to be particularly fit (or unfit) for purpose?

3. What, broadly, are the characteristics of high-quality (and low-quality) reviews undertaken by realist or meta-narrative methods? What can we learn from the best (and worst) examples so far?

4. What challenges have reviewers themselves identified (e.g. in the introduction or discussion sections of their papers) in applying these approaches? Are there systematic gaps between the 'theory' and the steps actually taken?

5. What is the link between realist and meta-narrative review and the policymaking process? How have published reviews been commissioned or sponsored? How have policymakers been involved in shaping the review? How have they been involved in disseminating and applying its findings? Are there models of good practice (and of approaches to avoid) for academic-policy linkage in this area?

6. How have front-line staff and service users been involved in realist and meta-narrative reviews? If the answer to this is 'usually, not much', how might they have been involved and are there examples of potentially better practice which might be taken forward?

7. How should one choose between realist, meta-narrative and other theory-driven approaches when selecting a review methodology? How might (for example) the review question, purpose and intended audience(s) influence the choice of review method?

The output of this phase will be a provisional summary organised under the above headings and highlighting for each question the key areas of knowledge, ignorance, ambiguity and uncertainty. This will be distributed to the Delphi panel as the starting-point for their guidance development work.

### Details of online Delphi process

We will follow an online adaptation of the Delphi method (see above) which we have developed and used in a previous study to produce guidance on how to critically appraise research on illness narratives [38]. In that study, a key component of a successful Delphi process was recruiting a wide range of experts, policymakers, practitioners and potential users of the guidance who could approach the problem from different angles, and especially people who would respond to academic suggestions by asking "so-what" questions.

Placing the academic-policy/practice tension central to this phase of the research, we hope to construct our Delphi panel to include a majority of experienced academics (e.g. those who have published on theory and method in realist and/or meta-narrative review). We will also hope to recruit policymakers, research sponsors and representatives of third sector organisations. These individuals will be recruited by approaching relevant organisations and email lists (e.g. professional networks of systematic reviewers, C.H.A.I.N., INVOLVE), providing an outline of the study and selecting those with greatest commitment and potential to balance the sample.

We will draw on our own experience of developing standards and guidance, as well as on published papers by CONSORT, PRISMA, AGREE, SQUIRE and other teams working on comparable projects [15,17,18,43].

The Delphi panel will be conducted entirely via the Internet using a combination of email and online survey tools. It will begin with a 'brainstorm' round ('round 1') in which participants will be invited to submit personal views, exchange theoretical and empirical papers on the topic and suggest items that might could be included in the publication standards. This will be done as a warm-up exercise and panel members will be sent our own preliminary summary (see above). These early contributions, along with our summary, will be collated and summarised in a set of provisional statements, which will be listed in a table and sent to participants for ranking ('round 2'). Participants will be asked to rank each item twice on a 9-point Likert scale (1 = strongly against to 9 = strongly in favour), once for relevance (i.e. should a statement on this theme/topic be included at all in the guidance?) and once for validity (i.e. to what extent do you agree with this statement as currently worded?). Those who agree that a statement is relevant but disagree on its wording will be invited to suggest changes to the wording. In this second round, participants will again be invited to suggest additional topic areas and items.

Each participant's responses will be collated and the numerical rankings entered onto an Excel spreadsheet. Median, inter-quartile and maximum-minimum range for each response will be calculated. Statements that score low on relevance will be omitted from subsequent rounds. Further online discussion will be invited on statements that score high on relevance but low on validity (indicating that a rephrased version of the statement is needed) and on those where there is wide disagreement about relevance or validity. Following discussion, a second list of statements will be drawn up and circulated for ranking ('round 3'). The process of collation of responses, further email discussion, and re-ranking will be repeated until maximum consensus is reached ('round 4' et seq.). In practice, very few Delphi panels, online or face to face, go beyond three rounds since participants tend to 'agree to differ' rather than move towards further consensus [38].

Residual non-consensus will be reported as such and the nature of the dissent described. Making such dissent explicit tends to expose inherent ambiguities (which may be philosophical or practical) and acknowledges that not everything can be resolved; such findings may be more use to reviewers than a firm statement which implies that all tensions have been "fixed".

### Preparing teaching and learning resources

A key objective of this study is to produce publicly accessible resources to support training in realist and meta-narrative review. We anticipate that these resources will need to be adapted and perhaps supplemented for different groups of learners, and interactive learning activities added [44]. Taking account of the format and orientation of other comparable materials (e.g. courses produced by the International Cochrane and Campbell Collaborations), though not necessarily aligning with these, we will develop and pilot draft learning objectives, example course materials and teaching and learning support methods. We will draw on our previous work on course development, quality assurance and support for interactive and peer-supported learning in healthcare professionals [35,44-46]

### Real-time piloting

The sponsor of this study, the National Institute for Health Research Service Delivery and Organisation (NIHR SDO) Programme, supports secondary research calls for rapid, policy-relevant reviews, some though not all of which seek to use realist or meta-narrative methods. We will work with a select sample of teams funded under such calls, as well as other teams engaged in relevant ongoing reviews (selected to balance our sample), to share emerging recommendations and gather real-time data on how feasible and appropriate these recommendations are in a range of different reviews. Over the 27-month duration of this study, we anticipate recruiting two cohorts of review teams over the course of this study: with the first cohort, we will use provisional standards, guidance and training materials based on our initial review of the literature. With the second cohort, we will pilot the standards, guidance and training materials which have been produced/refined via the Delphi process. After following two cohorts of review teams through their reviews, we will further revise the outputs as a master document before considering how to modify these for different audiences.

Training and support offered to these review teams will consist of three overlapping and complementary packages:

1. An 'all-comers' online discussion forum via Jiscm@il http://www.jiscmail.ac.uk/RAMESES for interested reviewers who are currently doing or have previously attempted a realist or meta-narrative review. This will be run via 'light-touch' facilitation in which we invite discussion on particular topics and periodically summarise themes and conclusions (a technique known in online teaching as 'weaving'). Such a format typically accommodates large numbers of participants since most people tend to 'lurk' most of the time. Such discussion groups tend to generate peer support through their informal, non-compulsory ethos and a strong sense of reciprocity (i.e. people helping one another out because they share an identity and commitment) [47] and they are often rich sources of qualitative data. We anticipate that this forum will contribute key themes to the quality and reporting standards and learning materials throughout the duration of the study.

2. Responsive support to our designated review teams. Our input to these teams will depend on their needs, interests and previous experience and hence is impossible to stipulate in detail in advance. In our previous dealings with review teams we have been called upon (for example) to assist them in distinguishing 'context' from 'mechanism' in a particular paper, extracting and formalising programme theories, distinguish middle-range theories from macro or micro theories, develop or adapt data extraction tools, advise on data extraction techniques, and train researchers in the use of qualitative software for systematic review.

3. A 'learning set' series of workshops for designated review teams. Much of the learning in such workshops is likely to come from the review teams themselves, and if participants are experienced and wish to offer teaching to others on particular relevant topics this will be encouraged. For the first workshop we will prepare a core syllabus of basic training oriented to explicit learning outcomes, delivered as a combination of prior self-study materials and short taught sessions on the day. Even at the first workshop, however, most of the time will be spent applying the basic principles to the real worked examples of reviews being undertaken.

As explained above, the first cohort of review teams will be run as a pilot and we will explain this to the participants, thereby gaining their active engagement in improving the programme for subsequent learners.

## Discussion

Realist and meta-narrative reviews are relatively new systematic review methods in health services research. They potentially offer great promise in unpacking the 'black box' of the many complex interventions that are increasingly being used to improve health and patient outcomes. As relatively experienced users of these methods, we have noted a number of common and recurrent challenges that face grant awarding bodies, peer-reviewers, reviewers and users. These centre on two closely related questions, namely how to judge if a realist or meta-narrative review, or a proposal for such a review, is of 'high quality' (including, for completed reviews, how 'credible' and 'robust' findings are) and how to undertake such reviews. Our experience to date suggests that we can go a long way towards answering these questions by giving due consideration to the theoretical and conceptual underpinnings of realist and meta-narrative reviews, outlined briefly below.

Realist review is based on a realist philosophy of science, which permeates and informs its underlying epistemological assumptions, methodology and quality considerations. Meta-narrative review takes a more constructivist philosophical position, though it is compatible with approaches which propose the existence of a social reality independent of our constructions of it. The meta-narrative approach seeks to tease out and explore the full range of philosophical positions represented in the primary literature.

One of the most common misapplications we have noted is that reviewers have not always appreciated the underlying philosophical basis of these review methods (and the implications of these for how the review should be conducted). Instead, they have based their reviews explicitly or implicitly on fundamentally different philosophical assumptions - most commonly the positivist notion that generalisable truths are best generated from controlled experiments, especially randomised trials.

Even when a realist philosophy of science has been adhered to in a realist review, reviewers - ourselves included - often struggle with recurring conceptual and methodological issues. 'Mechanisms' present a particular challenge in realist review - how to define them, where to locate them, how to identify them and how to test and refine them. Both review methods trade on the use of theoretical explanations to make sense of the observed data. Realist reviewers commonly grapple with how to define a theory (what, for example, is the difference between a 'programme theory' and a 'middle-range theory'?) and what level of abstraction is appropriate in what circumstances. On a more pragmatic level, those who seek to produce theory-driven reviews of heterogeneous topic areas wrestle with a broad range of 'how to' issues: how to define the scope of the review; how and to what extent to refine this scope as the review unfolds; what literature(s) to search and how; how to 'critically appraise' what is often a very diverse sample of primary studies; how to collate, analyse and synthesise findings; how to make recommendations that are academically defensible and useful to policymakers; and so on.

In conclusion, whilst realist and meta-narrative reviews hold much promise for developing theory and informing policy in some of the health sector's most pressing questions, misunderstandings and misapplications of these methods are common. The time is ripe to start on the iterative journey of producing guidance on quality and reporting standards as well as developing quality-assured learning resources to ensure that funding decisions, execution, reporting and use of these review methods is optimised. Acknowledging that research is never static, the RAMESES project does not seek to produce the last word on this topic but to capture current expertise and establish an agreed 'state of the science' on which future researchers will no doubt build.

The Delphi panel will commence in September 2011 and we anticipate that a paper describing the guidance will be submitted by September 2012. The online discussion forum is open to anyone with an interest in realist and meta-narrative reviews and may be found at http://www.jiscmail.ac.uk/RAMESES

## Competing interests

The authors declare that they have no competing interests.

## Authors' contributions

TG conceptualised the study with input from GWo and RP. TG wrote the first draft and GWo, GWe and RP critically contributed to and refined this manuscript. All authors have read and approved the final manuscript.

## Pre-publication history

The pre-publication history for this paper can be accessed here:

http://www.biomedcentral.com/1471-2288/11/115/prepub
